# Pattern of electrolyte imbalance in Type 2 diabetes patients: Experience from a tertiary care hospital

**DOI:** 10.12669/pjms.35.3.844

**Published:** 2019

**Authors:** Rashid Naseem Khan, Farhana Saba, Syedhh Fatima Kausar, Muhammad Hassan Siddiqui

**Affiliations:** 1 *Dr. Rashid Naseem Khan, MBBS, MCPS, MD. Principal, Consultant Physician, Liaquat College of Medicine and Dentistry and Darul Sehat Hospital, Karachi, Pakistan*; 2 *Dr. Farhana Saba, MBBS, FCPS. Internal Medicine Resident, Liaquat College of Medicine and Dentistry and Darul Sehat Hospital, Karachi, Pakistan*; 3 *Dr. Syedhh Fatima Kausar, MBBS, FCPS. Internal Medicine Resident, Liaquat College of Medicine and Dentistry and Darul Sehat Hospital, Karachi, Pakistan*; 4 *Dr. Mohammad Hassan Siddique, MBBS. House Officer, Liaquat College of Medicine and Dentistry and Darul Sehat Hospital, Karachi, Pakistan*

**Keywords:** Diabetes mellitus, Electrolyte imbalance, Hyperglycemia, Fasting blood glucose

## Abstract

**Objective:**

To determine frequency of electrolyte imbalance including sodium, chloride, potassium and magnesium levels present in patients with uncontrolled diabetes at a tertiary care hospital in Karachi.

**Methods:**

This was a descriptive cross sectional study conducted at Medicine Department, Darul Sehat Hospital, Karachi, Pakistan from March 2017 to October 2017. A total of one hundred and eighty one admitted and OPD patients with uncontrolled diabetes (HbA1c more than 7%) were included and their demographics, comorbidities, microvascular complications, drug history, fasting and random blood glucose and serum electrolyte levels were recorded.

**Results:**

In uncontrolled diabetes mellitus, decrease in serum sodium and chloride levels were observed to be statistically highly significant (p-value less than or equal to 0.05) while that of potassium and magnesium showed insignificant alterations. Sodium level was also observed to decline with increasing pattern of urine for microalbumin.

**Conclusion:**

Electrolyte imbalance is markedly present in patients with uncontrolled blood sugars therefore serum electrolytes should be routinely measured in patients with type 2 diabetes mellitus. Serum fasting blood glucose can be used as a predictor for electrolytes.

## INTRODUCTION

Diabetes is now one of the most common non-communicable diseases globally. The number of people with diabetes has risen from 108 million in 1980 to 422 million in 2014. The global prevalence of diabetes among adults over 18 years of age has risen from 4.7% in 1980 to 8.5% in 2014.^1^ Diabetes prevalence has been rising more rapidly in middle- and low-income countries. Diabetes is a major cause of blindness, kidney failure, heart attacks, stroke and lower limb amputation.^2^,^3^ In 2012, an estimated 1.5 million deaths were directly caused by diabetes and another 2.2 million deaths were attributable to hyperglycemia. Almost half of all deaths due to high blood glucose occur before the age of 70 years. WHO projects that diabetes will be the 7th leading cause of death in 2030. More than 425 million people have diabetes in the world and approximately 38.7 million people in the Middle East and North Africa Region; by 2045 this will rise to 82 million. There were 7.5 million cases of diabetes in Pakistan in 2017.^4^

Electrolytes play an important role in several body mechanisms, to name a few it helps maintain acid base balance, membrane potential, muscle contraction, nerve conduction and control body fluid. Alterations in electrolytes homeostasis may lead to physiologic disorders. Insulin has been shown to activate Na± /K± -ATPase enzyme. Therefore, low serum insulin level reduces Na± /K± -ATPase activity with poor Na± and K ± metabolism as a result and so transport across biomembranes as well as hindered monosaccharide uptake by intestinal epithelia occurs. In diabetes mellitus, hyperglycemia causes glucose induced osmotic diuresis with resultant loss of body fluids and electrolytes.^5^

Several studies have estimated the electrolytes levels in diabetes mellitus in several countries and showed the association between electrolytes and hyperglycemia.^5^,^6^ According to a study done by Javaid A et al they compared metformin, glibenclamide and combination of these two, to see the effect on electrolyte imbalance and found that low sodium and higher potassium was seen in all these cases with insignificant difference.^7^ In another study by Yasmin F et al, they found lower levels of all electrolytes i.e. sodium, potassium, chloride, calcium, magnesium and phosphorus.^8^ Since majority of these studies were done in western countries having different race and genetics and according to our knowledge only two of these studies^7^,^8^ have been done here in Pakistan, that’s why this study was conducted to look for electrolyte imbalance in cases of uncontrolled Diabetes Mellitus in our population.

## METHODS

A descriptive cross sectional study conducted at Darul Sehat Hospital, Karachi, Pakistan from March 2017-till October 2017. The sample size was calculated as 181 using the WHO sample size calculator by keeping the confidence interval equal to 95% and margin of error equal to 5.5% and anticipated prevalence of electrolyte imbalance as 18.7% in previous studies.^9^ The cases of both genders, age 30 years and above and having type 2 diabetes mellitus, with HbA1c of more than or equal to 6.5%,^10^ treated using different oral hypoglycemic agents and insulin regimens, for at least one year and more (assessed on history and medical records) were selected through non probability consecutive sampling from OPD and wards. Patients were labelled as uncontrolled diabetes with HbA1c of more than or equal to 7%.^11^ The data was collected by post graduate trainees and house officers of department of Internal Medicine through standardized proforma after taking informed consent from the patients. The variables includes the demographics of patients, age, gender, MR number, and duration of diabetes mellitus, height, weight, co-morbidities of the patients, diabetic neuropathy, retinopathy, electrolyte levels, urine for microalbumin, fasting blood glucose and medications history (calcium channel antagonists, insulin, beta blockers and oral hypoglycemic agents).

Diabetic neuropathy was assessed by 10-g Semmes-Weinstein Monofilament. It was assessed by the sensation of pressure using the buckling 10-g monofilament on particular sites of the foot and patient is asked to respond yes or no.^12^

Diabetic retinopathy was diagnosed with fundus examination, performed by an ophthalmologist. Patients irrespective of grade of retinopathy like macular edema, nonproliferative or proliferative diabetic retinopathy were labeled as having diabetic retinopathy. Diabetic nephropathy was assessed by measuring urine for microalbumin levels, microalbumin levels more than 30 mg in 24 hour urinary protein collection were labelled as having diabetic nephropathy.^13^ The exclusion criteria of this study was pregnant, handicapped, disabled patients, malabsorption syndrome, Cushing and Conn’s syndrome, Addison’s disease, type 1 diabetics, patients with renal or hepatic failure, connective tissue disorders, renal tubular acidosis and the patients already on diuretics, ACE inhibitors or ARBS and steroids. The study was approved by the Ethical committee of Liaquat College of medicine and dentistry and Darul Sehat Hospital.

Data were analyzed using SPSS version 16.0. The baseline qualitative parameters like age group, gender, co-morbidities like hypertension, with blood pressure more than 140/ 90 mmHg on two or more readings obtained on two or more occasions and ischemic heart disease assessed on history of coronary angiography revealing more than 50% narrowing in any one of the vessels. retinopathy, neuropathy, and other parameters were expressed as total number and percentages. Data for age, BMI (height in meters divided by weight in kilogram squared), electrolyte levels, and urine for microalbumin was expressed as mean with standard deviation and median with range values. Linear regression analysis was performed to estimate the electrolytes levels with the help of fasting blood glucose, model were also adjusted for age, gender and BMI. Scatter plot graphs were presented to show the trend between electrolytes and fasting blood glucose. The confounders were stratified for age, gender, BMI (kg/m^2^ where height will be calculated in meters by wall mounted scale and weight by electronic weighing machine in kilograms), hypertension and ischemic heart disease. Post-stratification relevant statistical tests were applied and p-values less than or equal to 0.05 were considered statistically significant.

## RESULTS

In the present study, 181 patients were involved, out of which 91 (50.3%) were female, 90 (49.7%) were male, with 131 (73.5%) people in age group range of 46- 75 years. Comorbidities were documented and 20 (11%) patients were hypertensive and 10 (5.5%) had hypertension with ischemic heart disease. Retinopathy was present among 16 (8.8%) patients, while 32 (17.7%) had neuropathy. Drug history revealed 21 (11.6%) were taking oral hypoglycemic agents, 5 (2.8%) calcium channel antagonists and 3 (1.7%) were on beta blockers.

The information on quantitative parameters of study is shown in Table-I. Results show that the mean age was 58.97±12.75 years, the mean for duration of diabetes was 10.6 ± 7.3. Mean BMI was calculated to be 25.46±5.61 kg/m2 and mean fasting blood glucose was 179.6 ± 77.6. Amongst electrolytes, mean serum sodium was 140.2 ± 6.1, mean serum potassium was 4.2 ± 0.53, while the mean chloride was 101.9 ± 5.9 and the mean magnesium was 1.9 ± 0.33.

**Table-I:** Baseline characteristics and parameter of study participants (n=181).

Characteristics	n(%)	Characteristics	n(%)
Gender		Neuropathy	
Male	90(49.7)	Present	32(17.7)
Female	91(50.3)	Absent	149(82.3)
Age Group		Oral Hypoglycemic Agents	
33 -45 years	28(15.5)	Yes	21(11.6)
46 - 75 years	133(73.5)	No	160(88.4)
>75 years	20(11.0)	Calcium Channel Antagonists	
Co-morbidity		Yes	5(2.8)
None	151(83.4)	No	176(97.2)
Hypertension	20(11.0)	Beta Blockers	
Hypertension and ischemic heart disease	10(5.5)	Yes	3(1.7)
Retinopathy		No	178(98.3)
Present	16(8.8)	Insulin	
Absent	165(91.2)	Yes	24(13.3)
		No	157(86.7)

*Parameters*	*Mean ± SD*	*Parameters*	*Mean ± SD*

Age (Years)	59 ± 12.8	FBS	179.6 ± 77.6
Duration of Diabetes (years)	10.6 ± 7.3	Sodium	140.2 ± 6.1
Weight	71 ± 17.6	Potassium	4.2 ± 0.53
Height	162.5 ± 10	Chloride	101.9 ± 5.9
BMI	25.5 ± 5.6	Magnesium	1.9 ± 0.33
Urine for microalbumin	42.3 ± 63		

**Fig.1:**
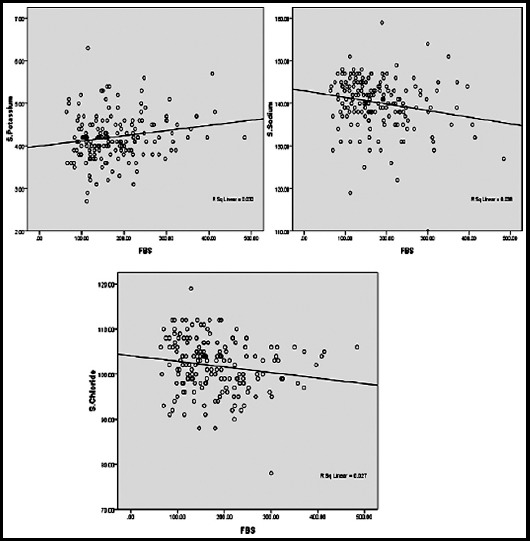
Scatter plot between FBS Serum Sodium, Potassium and chloride.

In this study, the regression model results with beta coefficients and with 95% confidence intervals, it was found that, fasting blood glucose significantly varied inversely with sodium, and chloride, while there was a positive correlation between serum potassium and fasting blood glucose, that a unit change increase in fasting blood glucose gives 0.02 average decline in sodium and chloride levels, and gives 0.01 unit increase in potassium. There was no significant association found with age, gender, comorbidities (hypertension and ischemic heart disease) and drug history. The model r-square were 3.3% for sodium, 2.6% for potassium, and 2.1% for chloride, magnesium did not have any significant effect on fasting blood glucose. Further model was adjusted for age, gender and BMI to see the effect of fasting blood glucose on electrolytes, results showed that serum sodium had significant inverse effect on fasting blood glucose in the presence of age, gender and BMI, however other electrolytes did not have any significant effect on fasting blood glucose (Table-II). Linear trend of electrolytes with fasting blood glucose is shown in Fig.1.

**Table-II:** Linear regression modeling for estimation of serum electrolytes using FBS.

Dependent Variables	Un-Adjusted Model	Adjusted Model**
Serum Sodium	-0.02* (-0.03,-0.01)	-0.03* (-0.04,-0.01)
Serum Potassium	0.01* (0.01,0.01)	0.01 (-0.01,0.01)
Serum Chloride	-0.02* (-0.03,-0.01)	-0.01 (-0.03,0.01)
Serum Magnesium	-0.02 (-0.03,-0.01)	0.01 (-0.01,0.01)

* p<0.05 was considered significant

** Model were adjusted for age, gender and BMI

Predictor: FBS

## DISCUSSION

Electrolyte imbalance is commonly present in patients with type 2 diabetes mellitus. The cause is usually multifactorial, but usually results from insulin deficiency in diabetic ketoacidosis and hyperglycemia.^14^ The present study showed significant reduction in serum sodium and chloride levels with increasing fasting blood glucose and increase in serum potassium levels. However, there was no significant change in serum magnesium levels.

This decreasing pattern of change in serum sodium levels as fasting blood glucose increases mainly confirmed previous observations, as shown by a study conducted in India, Parmar SK reported decrease in serum sodium and chloride levels as fasting blood glucose increased.^15^ Physiologically a well-known cause of dysnatremia in diabetes is osmotic diuresis. In patients with uncontrolled diabetes serum sodium levels vary, based on the balance between the hyperglycemia induced water movement out of the cells that lowers sodium and the glucosuria induced osmotic diuresis, which increases sodium.^14^ Elevations in blood glucose levels draws water out of the cells into extracellular place leading to hyponatremia.^16^

Serum sodium levels also showed inverse relationship with urine for microalbumin levels, suggesting that as renal function declines, serum sodium levels tend to decrease as well.^17^ Giacchetti et al demonstrated that activation of renal renin angiotensin system is responsible for the development of diabetic nephropathy, a major cause of end-stage renal disease.^18^ Hyponatremia was accompanied with hypochloremia in our study which was similar to the results of study done by Al Jameil N in Region of Saudi Arabia.^19^

Serum potassium levels were however not significantly affected by glycemic parameters in our study population, which is again in agreement with a study from region of Saudi Arabia that revealed insignificant changes in serum potassium levels in patients with both controlled and uncontrolled blood glucose levels.^20^ Although significant variations in serum potassium levels have been shown in other studies, Parmar SK et al and Saito et al reported a decreasing pattern in serum potassium levels with raised fasting blood glucose.^15^,^21^ Also serum magnesium levels remained normal in our study, whereas Arpaci D et al reported decreasing serum magnesium levels as HbA1c level increased.^22^

## CONCLUSION

Present study showed the importance of measuring serum electrolytes in patients with type 2 diabetes mellitus. As fasting blood glucose rises, electrolytes mainly sodium, chloride and potassium become more deranged significantly. Also, raised fasting blood glucose worsens renal function, as shown by an increase in microalbumin levels in urine. Serum sodium levels vary inversely with urine for microalbumin in our study and can be used as a predictor for nephropathy. Therefore measuring serum electrolytes in type 2 diabetes patients should be done as part of routine patient care.
